# Prolonged acyclovir therapy for Herpes simplex virus (HSV)-1–associated hepatitis in an immunocompetent man

**DOI:** 10.1016/j.idcr.2025.e02293

**Published:** 2025-06-19

**Authors:** Oulfa Boussetta-Charfi, François Duprey, Rémi Balluet, Marie-France Lutz, Sylvie Gonzalo, Anne-Camille Faure, Annie Evers, Sara Chenafi-Adham, Elisabeth Botelho-Nevers, Guillaume Dupont, Thomas Bourlet, David Boutolleau, Sylvie Pillet

**Affiliations:** a Laboratory of Infectious Agents and Hygiene, University Hospital of Saint-Etienne, France; b Research group, Clinique Mutualiste Saint-Etienne, Aesio group, Saint Etienne, France; c Laboratory of pharmacology-toxicology, University Hospital of Saint-Etienne, France; d Department of Infectiology, CHU St Etienne, France; e Laboratory of Hematology, University Hospital of Saint-Etienne, France; f AP-HP. Sorbonne Université, French National Reference Center for Herpesviruses (associated laboratory), Laboratory of Virology, Pitié Salpêtrière University Hospital, Paris, France

**Keywords:** Herpes simplex associated hepatitis, Liver failure, Intravenous acyclovir treatment, Nephrotoxicity, Viral load

## Abstract

Herpes simplex virus (HSV)-associated hepatitis (HH) has rarely been reported in immunocompetent patients. In the absence of mucocutaneous lesions and because of nonspecific biological features such as hepatic cytolysis, its diagnosis can be missed, leading to delayed acyclovir initiation and potentially poor outcomes. We report a case of a 62-year-old immunocompetent man who developed severe HH following a primary HSV-1 infection, diagnosed by a very high plasma HSV-1 DNA load. His condition was complicated by macrophage activation syndrome, which was managed using chemotherapy and corticosteroids. Acyclovir therapy (10 mg/kg every 8 ,h) was extended to Day 74 to a persistently detectable plasma HSV-1 DNA load, despite the normalisation of liver function tests. However, the optimal duration of antiviral therapy for HH remains unclear, as prolonged treatment may increase the risk of nephrotoxicity, whereas premature discontinuation may lead to fatal outcomes. Overall, this case illustrates that discontinuation of acyclovir did not result in a rebound of HSV-1 viraemia despite the persistent detection of low viral DNA load. Clinical and biological resolution of hepatitis may be helpful in guiding the decision to discontinue antiviral therapy. This case highlights the importance of early molecular diagnosis in atypical presentations of HH and contributes to guiding management strategies, particularly regarding antiviral treatment duration.

## Highlights

• Herpetic hepatitis (HH) with HSV is rare in immunocompetent patients.
• We report a severe HH case after HSV-1 infection in an immunocompetent man.
• He showed a persistent HSV-1 DNA load despite normal liver function tests.
• Therefore, acyclovir therapy was extended.
• Stopping acyclovir did induce HSV-1 viremia despite persistent viral DNA detection.

## Introduction

Herpes simplex virus (HSV) infection typically causes vesicular lesions on the skin or mucosa that are generally benign and self-limiting [1]. HSV establishes lifelong latency following primary infection, similar to other members of the *Herpesviridae* family, with potential reactivation triggered by various stimuli or by immunosuppression. In rare cases, HSV can cause severe complications, including hepatitis [2]. As of 2018, 137 cases of HSV-associated hepatitis (herpetic hepatitis, HH) had been reported, most of which occurred in immunocompromised patients and pregnant women [2]. However, few cases have been reported in immunocompetent, non-pregnant patients [3], [4], [5], [6], [7]. Without antiviral treatment, HH may rapidly progress to acute liver failure in 74 % of cases, with mortality rates reaching up to 90 % [8]. The reported predictors of mortality and the need for liver transplantation include advanced age, male sex, immunosuppression, marked hepatic cytolysis, disseminated intravascular coagulation, and encephalopathy [9]. Here, we present a case of severe HH following a primary HSV-1 infection in an immunocompetent man.

## Case presentation

This study was conducted in accordance with the ethical principles of (Declaration of Helsinki). This report conforms to the ethical approval standards currently applied in France. The patient approved the publication of his clinical and biological data.

A 62-year-old man was admitted to the emergency department with suspected right-sided pneumonitis. He had no mucocutaneous lesions, no known infectious contact, no recent travel, no unusual food intake, and no exposure to wild or non-domesticated animals. His medical history included type 2 diabetes managed with insulin and metformin, hypertension treated with a combination of perindopril and indapamide, and hypercholesterolaemia managed with atorvastatin. Upon arrival at the emergency room, the patient’s temperature was 40°C and blood pressure was 133/88 mmHg. Clinical examination revealed polyarthralgia and cutaneous hyperesthesia.

Laboratory investigations revealed marked hepatic cytolysis, with aspartate aminotransferase (AST) at 2604 international units (IU)/L (normal range [NR]: 10–50 IU/L), alanine aminotransferase (ALT) at 1537 IU/L (NR: 10–50 IU/L), C-reactive protein at 244 mg/L (NR: <5 mg/L), and lactate dehydrogenase at 2276 IU/L (NR: 135–250 IU/L). No evidence of cholestasis or jaundice was found. Coagulation tests were all within normal limits. Blood counts revealed thrombocytopenia (108,000/mm³; NR: 140,000–440,000/mm³) with normal haemoglobin levels (144 g/L; NR: 140–179). The total white blood cell count was slightly decreased at 3.9 Giga/L (NR: 4.0–11.0), associated with marked lymphopenia (0.199 Giga/L, NR: 1–4.8). Neutrophil count was normal (3.21 Giga/L; NR: 1.40–7.70), while eosinophil (0 Giga/L; NR: 0.02–063) and monocyte (0.70 Giga/L; NR: 0.18–1) counts were marginally decreased. The creatinine level was 102 µmol/L (NR: 62–106 µmol/L), and the urea level was 13.7 mmol/L (NR: 3.0–9.2 mmol/L), suggesting severe acute renal failure. No hepatotoxic drug was used; paracetamol level was checked and was within normal values (12.2 mg/L, NR: 10–30 mg/mL), and the clinical history ruled out other hepatotoxic medications. Computed tomography (CT) revealed hepatic steatosis but no signs of focal infection or adenopathy. Empirical treatment with doxycycline and cefotaxime was initiated.

As the liver function and acute kidney injury worsened, the patient was transferred to the intensive care unit (ICU). Blood cultures remained sterile. Human immunodeficiency virus (HIV) serology, including both antibodies and p24 antigen detection, was negative. The investigations thus focused on identifying the pathogens known to cause hepatitis. Serologic testing (IgG and IgM) was negative for hepatitis A, B, C, and E (HEV) viruses, as well as for *Coxiella burnetti*. HEV and *Leptospira* genomes were undetectable in the blood by quantitative PCR. In addition to classic hepatitis viruses (A – E), herpesviruses are also known to cause hepatitis and were tested. Serology indicated past immunity to both varicella-zoster virus and Epstein-Barr virus, the latter confirmed by the presence of IgG against Viral Capsid Antigen (VCA) and Epstein-Barr Nuclear Antigen (EBNA) without IgM. The patient was seronegative for both HSV-2 and cytomegalovirus. Given the absence of other identified pathogens, HSV-1 and HSV-2 genomes were tested in peripheral blood samples, which revealed a very high HSV-1 DNA load (2146,508 copies/mL). HSV-1 serology was negative for both IgG and IgM, indicating a primary infection. Therefore, intravenous acyclovir was initiated on ICU day 2 (D2) at a dose of 10 mg/kg every 8 h (full dose, not adapted to renal function).

**Fig. 1:**
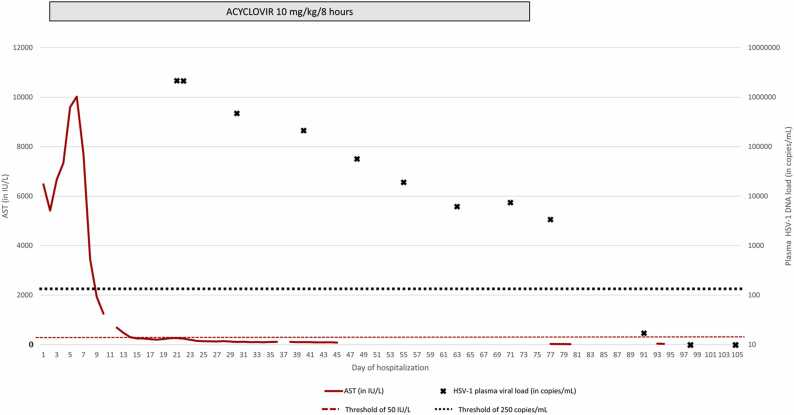
Evolution of aspartate aminotransferase (AST) levels and plasma HSV-1 DNA load (measured using the HSV1/2 ELITe MGB® Kit from BeGenius, Elitech, Torino, Italy) from emergency department admission (day 0) to day 105 of hospitalisation in the intensive care unit (ICU).

On D3, the patient developed macrophagic activation syndrome. This was characterized by thrombocytopenia (thrombocytes, 45 Giga/L) and neutropenia (neutrophils, 0.87 Giga/L), along with hyperferritinaemia (ferritin levels, 38,982 µg/L; NR: 30–400 µg/L) and hypertriglyceridemia (triglycerides, 3.32 g/L; NR: < 1.7 g/L). A myelogram smear revealed rare hemophagocytic figures involving all three myeloid lineages. Therefore, treatment with etoposide and corticosteroids was initiated and continued for 27 days. The complete blood count was normalised by D36, whereas the liver function tests returned to normal by D61. However, HSV-1 DNA in plasma remained detectable on D60 (Fig. 1), prompting genotyping resistance testing at the French National Reference Centre for Herpesviruses. No resistance mutations were found in the HSV-1 thymidine kinase or DNA polymerase genes. Additionally, the acyclovir plasma trough concentration was within the expected therapeutic range (1 mg/L, NR: 0.7–1 mg/L). Considering the improvement in liver function and decreasing HSV-1 viral load, acyclovir was discontinued on D74 (Fig. 1). On D87, the viral load was still detectable but below the limit of quantification (250 copies/mL) and became undetectable on D94.

The patient’s creatinine level rose at the beginning of hospitalisation, peaked at 498 µmol/L on D4, and gradually decreased after intermittent dialysis. A renal biopsy performed on D69 revealed acute tubular necrosis and approximately 30 % glomerular destruction, leaving the potential for renal recovery uncertain. After 99 days, the patient remained on intermittent dialysis, with no prospect of discontinuation.

Finally, the patient’s hospital stay was complicated by chronic renal failure necessitating haemodialysis; the patient also developed ICU neuropathy. At 9 months post follow up, he was still in the rehabilitation department.

## Discussion

HH is an uncommon complication of HSV infection that often progresses to acute liver failure [9]. HH typically occurs in pregnant women during the third trimester, as well as in solid organ transplant recipients and children [9]. However, 24 % of HH cases are reported in immunocompetent patients [9]. Male sex, advanced age, immunocompromised status, high transaminase levels, coagulopathy, and encephalopathy are poor prognostic factors associated with death or the need for liver transplantation [9]. Notably, the patient described in this report presented with three of these factors, suggesting a poor prognosis from the time of diagnosis.

As in the present case, mucocutaneous lesions are rarely present, making the diagnosis difficult to consider. Additionally, approximately 90 % of patients with HH exhibit an "anicteric hepatitis" profile, with normal or low bilirubin levels; however, a 100- to 1,000-fold increase in transaminase levels is observed [10]. Hepatic cytolysis caused by HSV is greater than that observed with other viruses in the *Herpesviridae* family [2]*.*

The reference test is based on histological analysis of a liver biopsy [11]; however, the procedure is invasive and was not performed in this case. HH can be diagnosed based on serology combined with HSV-1 DNA quantification in the blood, clinical symptoms, and liver function abnormalities. The appropriate treatment is intravenous acyclovir at a dose of 10 mg/kg every 8 h. The earlier treatment is initiated, the better is the outcome [12], especially because HSV resistance to acyclovir is rare (less than 1 % of immunocompetent patients) [13].

Acyclovir is excreted in the urine by glomerular filtration and tubular secretion, with an elimination half-life ranging from 2–3 h and up to 20 h depending on renal function [14]. The main mechanisms of nephrotoxicity include intratubular crystal deposition and direct tubular toxicity. Prolonged therapy and concomitant use of nephrotoxic drugs increase the risk of developing acyclovir-induced nephrotoxicity [15]. In this case, the residual acyclovir level was within the normal range (1 mg/L; NR: 0.7–1 mg/L) even though the dose was not adapted to the renal function, and renal biopsy ruled out acyclovir-associated nephropathy.

The duration of acyclovir treatment for HH is not well established. It is generally around 21 [2–73] days at a dose of 10 mg/kg every 8 h. As the patient received chemotherapy and corticosteroid therapy the day after acyclovir initiation, this may explain the slow decline in the HSV-1 DNA load in the blood. Considering the improvement in liver function, acyclovir was discontinued on D74, and the viral load was monitored weekly. Liver function remained normal, and the viral load was undetectable by D94.

In conclusion, the duration of acyclovir treatment for HH should be based on multiple factors, including the presence of HSV DNA in the blood, normalisation of hepatic transaminases, and clinical recovery. Thus, a persistent low-level HSV DNA load in the blood likely reflects the slow clearance of a large amount of viral genome (over 2 million copies/mL) from the bloodstream, rather than ongoing viral damage to the liver. Overall, suitable guidelines need to be established for the therapeutic management of HH. They should be based on prospective studies, biomarker-based decision-making, or longer-term follow-up.

## Ethical approval and Consent

This study was conducted in accordance with the ethical principles of (Declaration of Helsinki). This report conforms to the ethical approval standards currently applied in France. The patient approved the publication of his clinical and biological data.

## CRediT authorship contribution statement

**Sylvie Pillet:** Writing – review & editing, Writing – original draft, Validation, Supervision, Methodology. **François Duprey:** Writing – review & editing, Data curation. **David Boutolleau:** Writing – review & editing, Supervision, Methodology. **Oulfa Boussetta-Charfi:** Writing – original draft, Methodology, Formal analysis, Data curation, Conceptualization. **Lutz Marie France:** Writing – review & editing, Investigation, Conceptualization. **Balluet Remi:** Writing – review & editing, Methodology, Data curation. **Elisabeth Botelho-Nevers:** Writing – review & editing, Supervision. **Sara Chenafi-Adham:** Writing – review & editing, Validation. **Thomas Bourlet:** Writing – review & editing, Supervision. **Guillaume Dupont:** Writing – review & editing, Validation. **Sylvie Gonzalo:** Writing – review & editing, Validation. **Annie Evers:** Writing – review & editing, Validation. **Faure Anne Camille:** Writing – review & editing, Validation.

## Authors' contributions

Oulfa Boussetta-Charfi and Sylvie Pillet conceived and designed the study. All authors contributed substantially to the acquisition, analysis, and interpretation of the data. Oulfa Boussetta-Charfi drafted the article. All authors revised the article critically for important intellectual content. Sylvie Pillet reviewed and edited the final manuscript. All authors provided final approval for the submitted version of the article.

## Funding

This research did not receive any specific grants from funding agencies in the public, commercial, or not-for-profit sectors.

## Declaration of Generative AI and AI-assisted technologies in the writing process

During the revision of this work, the author(s) used ChatGPT Enterprise (chatgpt.com) to revise the entire manuscript and improve the grammar and sentence structure for better clarity and conciseness. After using this tool, the authors reviewed and edited the content as needed and take full responsibility for the content of the publication.

## Declaration of Competing Interest

The authors declare the following financial interests/personal relationships which may be considered as potential competing interests: Sylvie Pillet reports was provided by University Hospital of Saint-Etienne. If there are other authors, they declare that they have no known competing financial interests or personal relationships that could have appeared to influence the work reported in this paper.

## Data Availability

The datasets generated and/or analysed during the current study are not publicly available owing to their personal nature (confidential medical file) but are available from the corresponding author upon reasonable request.

## Glossary

EBNA: Epstein-Barr Nuclear Antigen
HH: Herpes simplex virus-associated hepatitis
HSV: Herpes simplex virus
NR: normal range
VCA: Viral Capsid Antigen
